# From statistical inference to a differential learning rule for stochastic neural networks

**DOI:** 10.1098/rsfs.2018.0033

**Published:** 2018-10-19

**Authors:** Luca Saglietti, Federica Gerace, Alessandro Ingrosso, Carlo Baldassi, Riccardo Zecchina

**Affiliations:** 1 Microsoft Research New England, Cambridge, MA, USA; 2 Italian Institute for Genomic Medicine, Torino, Italy; 3 Politecnico di Torino, DISAT, Torino, Italy; 4 Center for Theoretical Neuroscience, Columbia University, New York, USA; 5 Bocconi Institute for Data Science and Analytics, Bocconi University, Milano, Italy; 6 Istituto Nazionale di Fisica Nucleare, Torino, Italy; 7 International Centre for Theoretical Physics, Trieste, Italy

**Keywords:** associative memory, attractor networks, learning

## Abstract

Stochastic neural networks are a prototypical computational device able to build a probabilistic representation of an ensemble of external stimuli. Building on the relationship between inference and learning, we derive a synaptic plasticity rule that relies only on delayed activity correlations, and that shows a number of remarkable features. Our *delayed-correlations matching* (DCM) rule satisfies some basic requirements for biological feasibility: finite and noisy afferent signals, Dale’s principle and asymmetry of synaptic connections, locality of the weight update computations. Nevertheless, the DCM rule is capable of storing a large, extensive number of patterns as attractors in a stochastic recurrent neural network, under general scenarios without requiring any modification: it can deal with correlated patterns, a broad range of architectures (with or without hidden neuronal states), one-shot learning with the palimpsest property, all the while avoiding the proliferation of spurious attractors. When hidden units are present, our learning rule can be employed to construct Boltzmann machine-like generative models, exploiting the addition of hidden neurons in feature extraction and classification tasks.

## 1. Introduction

One of the main open problems of neuroscience is understanding the learning principles which enable our brain to store and process information. Neural computation takes place in an extremely noisy environment: experiments show that various sources of variability and fluctuations make neurons, synapses and neural systems intrinsically stochastic [1]. Such internal noise can originate at different levels, for instance, from the unreliable transmission of synaptic vesicles, from the random opening and closing of ion channels or from the trial-to-trial variability in neural responses to external stimuli [2–6]. At the same time, even the typical sensory input is often blurry and ambiguous. A probabilistic inference framework is thus the natural choice for modelling all the uncertainties affecting neural learning [7].

A widespread belief is that learning occurs at the synaptic level, both in terms of creation of new connections and by synaptic strength potentiation or depression [8–10]. Synaptic plasticity can be encoded in a learning principle that relates the modulation of the efficacy of a synapse to its pre- and post-synaptic neural activity. The simplest synaptic plasticity rule, Hebb’s rule, states that positive correlation between pre- and post-synaptic spikes leads to long-term potentiation, while negative correlation induces long-term depression. One important feature of Hebbian plasticity is its capability to shape the connectivity of neural architectures in a way that captures the statistics of the stimuli. This issue has been addressed in a number of modelling studies, starting from the classical theory of development of neural selectivity [11], to more modern accounts of neural tuning that use homeostasis-stabilized Hebbian plasticity in large spiking network models [12].

On the other hand, it has long been recognized that Hebbian plasticity is capable of generating attractor dynamics in a variety of recurrent architectures: the concept of attractor neural network is one of the most important in modern neuroscience, in that it can account for a variety of neurophysiological observations of persistent activity in various brain areas. Examples include line attractor (neural integrator) models in oculomotor control [13], ring attractor models in head direction systems [14] and a plethora of models of persistent neural activity whose common feature is a local connectivity pattern which stabilizes bump attractors by means of lateral inhibition.

The main intuition that led to the introduction of the prototypical model of attractor network—the Hopfield model—was that the *frustration phenomenon* in disordered systems (spin glasses), namely the proliferation of metastable states due to the strongly heterogeneous nature of the couplings, could be exploited to embed uncorrelated patterns as steady states of a network dynamics. In the Hopfield model, a straightforward application of Hebb’s rule leads to a definition for the synaptic weights that allows for an extensive number of attractors to be stored, but exhibits a phenomenon known as *catastrophic forgetting* [15]: all memories are lost, due to the existence of an absorbing spin glass state uncorrelated with the memories, as soon as the maximum number of attractors is exceeded. Since the original introduction of the Hopfield model [16], many generalized Hebb rules have been proposed, able to deal with sparse patterns or low activity levels (e.g. [15]). Moreover, Hebbian learning has been profitably used to embed attractor states in a variety of neural network models spanning from binary units to graded neurons (rate models) [17] and spiking networks [18].

Different lines of research concerning attractor neural networks in statistical mechanics and computational neuroscience have strong ties with the study of generative energy-based models: the formalism of Boltzmann machines allows for a generalization to neural networks with hidden neural states [19,20]. This introduction, though, comes at the price of serious technical complications in the definition of a viable learning rule. Some of these models have become popular also in the machine-learning community, after proving themselves as useful tools in several deep-learning applications. This stimulated the development of various learning heuristics, the most renown being contrastive divergence (CD) [21], and inference methods [22–25].

An alternative direction of research is motivated by many inference problems in biological systems, where couplings are typically asymmetric and possibly time-varying. The study of the dynamics and learning in these purely kinetic models is complicated by the lack of analytical control over the stationary distribution [26–28]: a number of interesting mean field techniques based on generalization of Thouless–Anderson–Palmer (TAP) equations have been proposed [26–28] in this context.

In this study, we approach many of these problems from a unified perspective: the main goal of the paper is to devise a biologically plausible learning rule which could allow a general stochastic neural network to construct an internal representation of the statistical ensemble of the stimuli it receives. In the following, we consider the case of asymmetric synaptic couplings and derive a learning scheme in which the updates involve only purely local and possibly noise-affected information. The proposed plasticity rule does not rely on the presence of supervisory signals or strong external stimuli, and proves to be compatible with Dale’s principle, which requires the homogeneity of the neurotransmitters released by one neuron across its synaptic terminals [29,30]. In this work, we define the learning process in an online context, and our analysis will be restricted to the case of discrete time dynamics.

In the Results section, for clarity of exposition, we will mostly focus on the specific case of fully visible neural networks, giving only a brief overview on the extension to networks comprising hidden neurons. This last setting is largely expanded upon in the electronic supplementary material, where we also provide further analytical insights and the implementation details of our numerical experiments.

## 2. Results

We present our main results in three different subsections. In the first one (The model), we derive the new plasticity rule in a framework that encompasses a wide variety of unsupervised and semi-supervised problems, such as the construction of attractor networks and learning in generative models with more complicated structures. In the second one (Fully visible case), we specialize to the case of attractor networks containing only visible neurons. After describing a link with the maximum pseudo-likelihood method, we study the numerical performance of the new learning rule in various settings, showing how it deals with finite external fields, different coding levels and the constraint of Dale’s Law. We then test the rule in the case of correlated memories, we investigate its proneness to create spurious attractors and we measure its palimpsest capacity. Finally, in the third section (Adding hidden neuronal states), we give an introduction to the more complex case of stochastic networks with hidden neurons, and review some of the results, presented in the electronic supplementary material, section *VI*, that were obtained in this setting.

### 2.1. The model

We consider the customary simple set-up of a network of *N* stochastic binary neurons 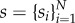, with each 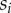 either in 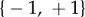 or 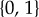, connected by a set of asymmetric synaptic weights 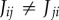, which evolves with a discrete-time synchronous dynamics described by the Glauber transition probability: the next state 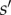 of the system depends on the current state *s* according to the following factorized probability distribution:2.1
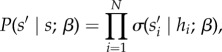
with 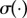 being a sigmoid-shaped neural activation function defined by 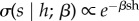 (the proportionality constant being set by normalization), and 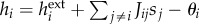 being the total neural current—or *local field*—obtained by adding up the recurrent contributions from other neurons to the external stimulus 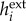. The quantity 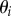 serves as a local threshold. The dynamics of the system is thus stochastic, and the parameter 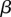 (which has the role of an inverse temperature in analogous physical models) provides a measure of the dynamical noise in the system. When the synaptic couplings *J* are finite and the external fields are time independent, the dynamics is known to be ergodic and a steady state defined by a unique stationary distribution is approached [26]. However, the analytical form of this steady state distribution is not known for general asymmetric kinetic models of the type we consider here.

In the following, we formulate the problem of learning as an unsupervised task where the network has to adapt its parameters in accordance with some plasticity rule: the goal is to learn an internal representation of a target probability distribution, which is to be inferred from a set of external stimuli conveyed to a subset of the neurons. Suppose we are given a time-independent binary pattern, a vector 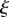 of length 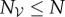 with components 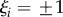, to be learned by the neural network. This pattern is presented to a group 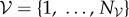 of ‘visible’ neurons in the form of an external field of variable intensity 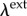 in the direction of 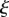, i.e. 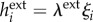 for 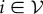, while the complementary subset 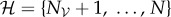 of ‘hidden’ neurons receive no external input. We want to model the scenario in which the stimulus intensity is high (although not as large as to clamp the neurons) at the onset, and rapidly vanishes. The initial presence of the field biases the dynamics of the system; in the retrieval phase, if the stimulus 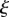 is sufficiently close to a pattern 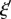 that the network has learned, the stationary probability distribution of the visible neuronal states should get focused in the direction of 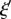 even after the stimulus is no longer present.

For the sake of comparison, the classical Hopfield network with Hebbian learning can be framed in the same setting, as follows: we assume that there are no hidden neurons, and the dynamics of the stimulus presentation is a simple two-step process in which the stimulus intensity 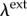 is initially effectively infinite (such that the other components of the inputs become irrelevant and the dynamics of the neurons becomes deterministic and fixed, i.e. such as to clamp the network) and then drops to 0. The learning rule in that case actually only uses the information about the state of the network during the clamped phase: 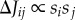 where 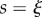 as a consequence of the clamping. In the retrieval tests, the clamped phase is used to initialize the network, which subsequently evolves by its own internal dynamics in absence of further stimuli.

In our framework, we exploit the dynamics of the stimulus during the learning phase, extracting the correlations that the stimulus induces on the network dynamics and using them to train the network: since the final goal of the network is to learn from the driving effect of the external field, we may require the dynamical evolution in the freely evolving network to maximally resemble the stimulus-induced evolution. Intuitively, this amounts at training the network to compensate the gradual vanishing of the external field by adapting its own recurrent connections. This requirement can be framed formally as the minimization of a Kullback–Liebler (KL) divergence between two different conditional probability distributions corresponding to different levels of intensity of the external field, 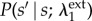 and 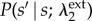, averaged over some initial state probability distribution 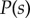. The analytical details can be found in the electronic supplementary material, section *II*. As explained in more detail below, the distribution 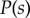 is supposed to be concentrated (for the visible part of the network) around the direction of the pattern 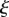, such that 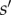 will also be concentrated around 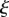 as the combined effect of the initial conditions, the external field and of the recurrent connections; when the effect of the external field decreases, the recurrent connections will tend to compensate for this. If these conditions can be met, then the procedure can be applied repeatedly.

As an initial simplified case, consider the same setting as the Hebbian learning, i.e. the limiting case of an infinite 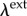, in which the visible part of the network dynamics is clamped. The *stationary* probability distribution can thus be factorized over the visible neuronal states 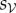:2.2
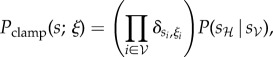
where 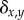 denotes the Kronecker delta symbol which equals 1 if 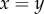 and 0 otherwise. Here, the conditional probability of the hidden neuronal states 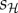, given the visible, cannot be written explicitly without losing generality. In our learning scheme, we seek to minimize the difference between the initial (fully clamped) situation and the subsequent zero-field situation; this requirement produces the following simple learning rules for the synaptic couplings and the thresholds:2.3
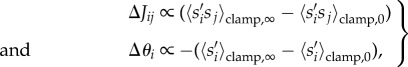
where *s* and 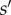 denote two successive states of the network, as above, and 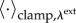 is defined as an average over the possible dynamical responses starting from a state sampled from 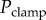:2.4
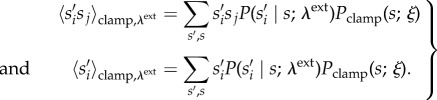
In the limiting case, we simply have 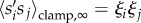 for the visible neurons. In general, however, efficiently obtaining an accurate estimate of this average can pose serious technical challenges.

**Figure 1.**
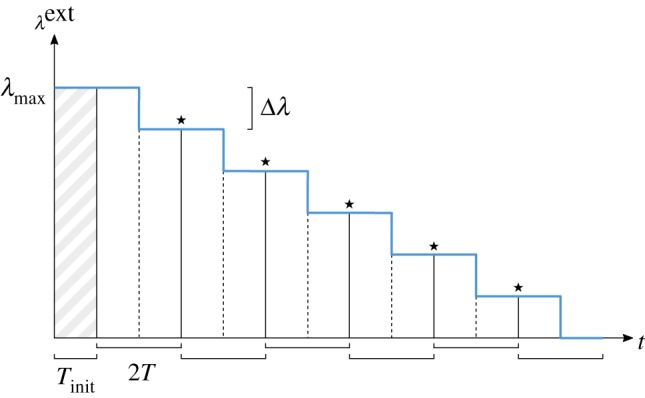
DCM learning protocol scheme. This represents the learning process for one pattern presentation. The blue curve shows the stepwise dynamics of the external field 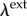 as a function of the time *t* of the network dynamics. The first time period (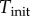 time steps, shaded) serves for initializing the network state in the proximity of the pattern. The protocol then proceeds in windows of 2*T* steps, each one divided in two phases. In the middle of each window, the field intensity drops by 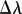. The time-delayed correlations are recorded separately for the two phases. The parameters are updated at the end of each window, in correspondence of the 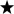 symbols, according to equation (2.6). (Online version in colour.)

Since the case of a clamping stimulus is biologically unrealistic, we explore a setting in which the amplitude of the external signal is comparable to the recurrent contribution exerted by the surrounding neurons: instead of trying to match the dynamical response of a clamped model with a freely evolving one, we introduce a learning protocol based on a time-dependent field intensity 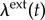, which decreases to zero starting from a finite initial value 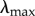. In the following, we will consider a staircase signal intensity 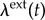, lowered by a fixed amount 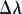 after every 2*T* steps of the time-discretized network dynamics (figure 1). We should remark, however, that the results presented hereafter are quite robust with respect to variations in the precise details of the dynamical protocol for the field, and that the above choice was purely made for simplicity of presentation and analysis.

The training protocol prescribes the network to try and match its dynamical behaviour at a given level of the field λ with that at a lower level 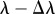, where the dynamical behaviour is measured in terms of the time-delayed correlations between neurons2.5
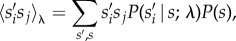
and 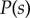 is some initial probability distribution roughly concentrated around the presented pattern 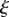 for the visible neurons. More precisely, we suppose that the overall distribution 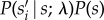 induces a dynamics which is confined around 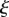 and ergodic within such region. When that is the case, sampling the temporal averages as the system evolves can provide an estimate of the averages involved in the above expression. It is reasonable to assume, and confirmed by our experiments, that this condition will be satisfied if the initial field 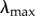 is sufficiently large, thus creating an effective basin of attraction, and if the system evolution manages to keep this confinement in place even when the field is decreased by adapting the recurrent connections.

Our learning protocol is thus defined as follows (figure 1): the network will first record for *T* time steps its time-delayed correlations at a given value of the field 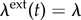; then, it will do the same for another *T* steps at a lower level, 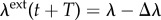, after which it will adjust its parameters such as to try to match the two sets of measurements (see below). The protocol will then restart with the same field 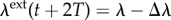 (but with updated network parameters), proceeding in this way until the field has dropped to zero. The network state is never reset during these steps; rather, it keeps following the dynamics of equation (2.1). An extra initial period of 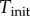 steps (we generally set 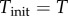 in our simulations) at 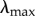 field is used to prepare the network and bias it in the direction of the pattern.

Therefore, in this approximation, we obtain a new plasticity rule (the notation 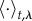 here denotes empirical averages over time in presence of a given field λ, and we switch to using *t* and 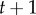 to denote two consecutive time steps):2.6
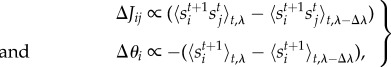
which simply tries to match the time-delayed correlations in the consecutive time windows, until the signal has vanished and the system evolves freely. All the needed information is thus local with respect to each synapse. In order to learn a given extensive set of *αN* patterns, the same procedure has to be repeated cyclically: a pattern is presented with decreasing intensity while the network adapts its parameters, then the network moves to the next pattern. The network is not reset even between one pattern and the next. We call this learning rule ‘delayed-correlations matching’, DCM for short. The full algorithm is detailed in the electronic supplementary material, section *IV*, together with the corresponding pseudo-code.

It is not necessary for the field dynamics to end up exactly at zero intensity: following the same idea proposed in [31], the learning scheme described above can be made more robust if one requires the network to face the presence of an antagonist field, that tries to interfere with the drawing effect of the basin of attraction. By considering a negative minimal intensity 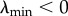, one can in fact both speed up the learning process and induce larger basins of attraction. If instead the aim is to learn new basins of attraction coherently, trying not to affect the previously stored memories, it can be useful to choose a positive 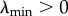: this ensures that the sampling process does not leave the neighbourhood of the presented pattern, risking ending up in a different memory and possibly deleting it (we will consider this prescription in the *one-shot learning* scenario).

### 2.2. Fully visible case

When a network with no hidden neurons is considered (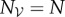), the learning problem effectively reduces to that of constructing a stochastic attractor neural network with binary units. Kinetically persistent neuronal states can be indeed observed even with asymmetric synaptic couplings *J*. We will require the network to embed as stable and attractive memories an extensive set of i.i.d. random binary ±1 patterns, denoted by 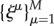, with 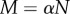 (each 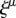 is an *N*-dimensional vector and *μ* represents a pattern index). The number of stored patterns per neuron *α* is the so-called *storage load* of the network.

Since the learning procedure is defined as a cyclical minimization of a KL divergence evaluated at the *M* patterns, the limiting case with just two dynamical steps and infinite initial field considered in equation (2.3) can here be reinterpreted exactly as an online optimization of the so-called log-pseudo-likelihood:2.7
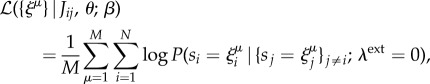
which is most frequently found in an inference framework [32,33], where the parameters of a generative model have to be inferred from a finite set of complete observations (see electronic supplementary material, section *II A*).

**Figure 2.**
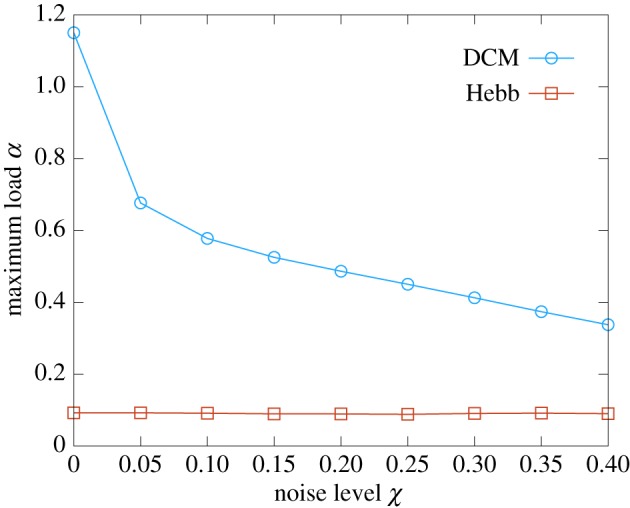
Maximum storage load as a function of the width of the basin of attraction for a network of *N* = 400 visible neurons. The red and blue curves show the results for the Hopfield model and the DCM rule, respectively. The noise level 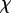 operatively measures the width of the basins of attraction (it is the fraction of corrupted bits that the network is able to correct, see the text for a more precise definition). Each curve is an average over 10 samples (error bars are smaller than point size). The inverse temperature parameter is set to 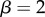 in order to fall within the retrieval phase of the Hopfield model. The critical capacity at zero temperature is lower than the Gardner bound, 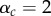, because of the stochastic component of the dynamics. (Online version in colour.)

In this case, the update for the synaptic couplings can be written more explicitly and allows for a clear comparison with the standard Hebbian plasticity rule:2.8
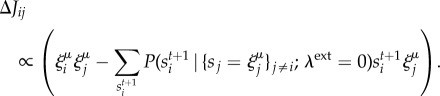
The DCM rule is explicitly asymmetric, and its differential form produces a homeostatic mechanism constantly trying to reproduce externally induced correlations in the network dynamics. While in the initial stages of the learning process the synaptic weights are modified according to a typical Hebbian prescription—potentiation in case of positive correlations and depression with negative ones—the comparator effectively avoids the possibly uncontrolled positive feedback loop of the Hebbian principle: no change in synapses will occur when the correlations in the absence of the stimulus already match the ones of the learned patterns. Incidentally, we also note that in the noise-free limit 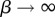 the perceptron learning rule is recovered (see electronic supplementary material, section *II B*). In the case of 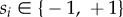 neurons, we studied numerically the trend of the maximum storage load achievable with the DCM rule as a function of the required width of the basins of attraction. We introduced an operative measure of the basin size, relating it to the level of corruption of the memories before the retrieval: a set of 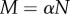 patterns is considered to be successfully stored at a noise level 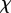 if, initializing the dynamics in a state where a fraction 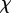 of the pattern is randomly corrupted, the retrieval rate for each pattern is at least 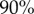 (for additional details, see the electronic supplementary material, section *IV A*). In figure 2, we compare the DCM rule with the Hopfield model, which is known to achieve a maximum storage load of approximately 0.14*N*.

**Figure 3.**
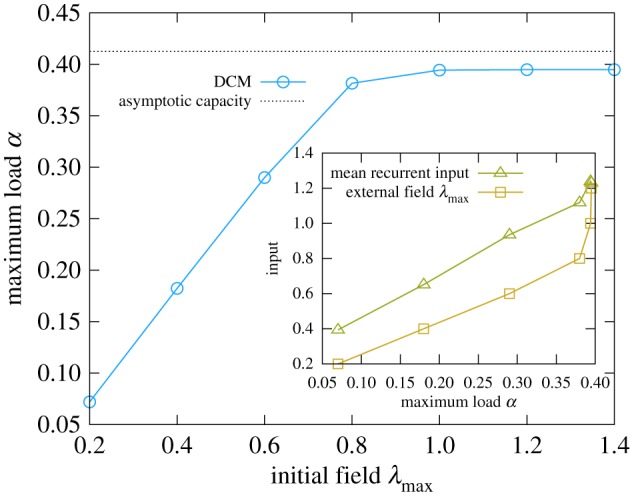
Maximum storage load as a function of the field intensity for a network of *N* = 400 neurons. The correlations were recorded in windows of *T* = 20 time steps and the field intensity step was 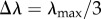. The noise level in the retrieval phase is set to 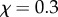 and the temperature to 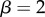. The curve was obtained by averaging over 100 samples. The inset shows a comparison between the recurrent and external components of the inputs, for the same data points of the main panel. The mean recurrent input was computed as the 2-norm of the mean values. This shows that the DCM rule is effective even for relatively small stimuli. (Online version in colour.)

If we move to the more biologically plausible scenario of finite time-dependent external fields (equation (2.6)), we clearly see in figure 3 that an infinite signal is actually redundant. If the external field intensity is high enough, the recorded time-delayed correlations carry enough information about the pattern to be learned. If instead the signal component in the local field is dominated by the recurrent contribution from other neurons the dynamics becomes completely noisy. Since the average strength of the connections between the neurons increases with the number of stored memories, the maximum storage load grows with the signal amplitude. Nevertheless, the results of pseudo-likelihood are already almost saturated at small field intensities 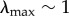, and the DCM rule generally works well even when the stimulus intensity is relatively small compared with the total recurrent input (see inset of figure 3). The implementation details are described in the electronic supplementary material, section *IV* .

**Figure 4.**
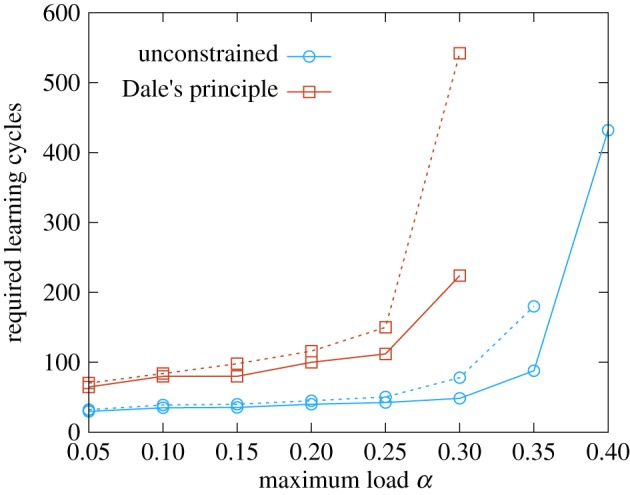
Required learning cycles as a function of the storage load, for unconstrained and constrained synapses for networks of size *N* = 200 (dashed curves) and *N* = 400 (full curves). The results for the case of unconstrained synapses (blue curves) and that of synapses satisfying Dale’s principle (red curves) are compared. Here the chosen inhibitory scheme is the soft ‘winner takes all’ mechanism. The noise level in the retrieval phase was set to 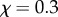, while the sparsity was fixed at 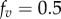 in order to avoid finite size effects with the relatively small networks. The curves are interrupted at the value of *α* where the algorithm starts failing. (Online version in colour.)

We also considered an alternative model with somewhat more biologically plausible features, using 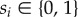 neurons (see the electronic supplementary material, section *I*) and sparse 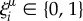 patterns, and forcing the synapses to satisfy Dale’s Law. This means that two sub-populations of excitatory and inhibitory neurons should be defined, the sign of their outgoing synapses being fixed *a priori*. Note that this restriction reduces the theoretical maximum capacity of the network, although not dramatically (roughly by half [34]). For simplicity, we restricted our analysis to the case where only excitatory synapses are plastic and a separate inhibitory sub-network provides a feedback regulatory effect, whose goal is to maintain the network activity 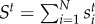 around a desired level 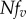 (the same sparsity level as the learned patterns), and preventing epileptic (all-on) or completely switched off states. We tested three different effective models that implement an inhibitory feedback mechanism: (i) a generalization of the global inhibitory mechanism described in [34], tuned such as to counterbalance the oscillations of the network activity around the desired level; (ii) a soft ‘winner-takes-all’ mechanism, effectively playing the role of a global inhibitory unit [35–45]; and (iii) a model with adaptive thresholds that allow the Dale’s principle model to behave approximately like the unconstrained one. The details, including the derivation of the parameters of all these schemes, are reported in the electronic supplementary material, section *III*. For all of them, the results are comparable to the ones shown in figure 4.

#### 2.2.1. Comparison with Hebbian plasticity rule

**Figure 5.**
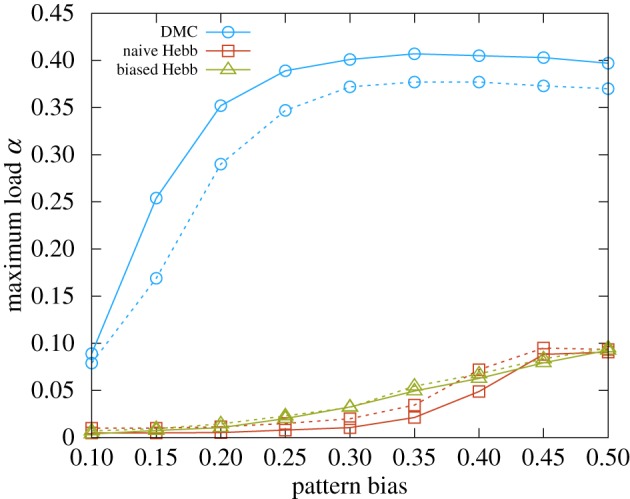
Maximum storage load as a function of the bias in the distribution of the patterns for networks of size *N* = 200 (dashed curves) and *N* = 400 (full curves). The correlation is introduced trivially: each pattern is built by extracting spins from a biased distribution 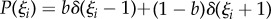. The blue curves show the scaling properties of the capacity of the DCM rule as a function of the bias. The drop in the performance for small biases is due to finite size effects, and the performance improves with *N*. The red and green curves show the results for the naive Hebb rule and the generalized Hebb rule adapted to the biased case, respectively (see the electronic supplementary material, section *IV D*). For larger *N*, the capacity for all unbalanced cases is expected to drop to 0. All the curves were obtained by averaging over 10 samples (error bars are smaller than the point size). (Online version in colour.)

Most real-world data are inherently sparse and redundant, so that it is crucial for a plasticity rule to be able to deal with a pattern set exhibiting internal correlations. The most trivial way of introducing a positive correlation among the patterns is to bias the probability distribution from which the patterns are extracted, i.e. using the probability distribution 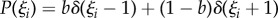 for the pattern components, with 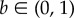 (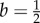 being the unbiased case). The Hebbian learning rule needs to be adapted for enabling learning of biased patterns [46] (see the electronic supplementary material, section *IV D*), and the modification requires explicit knowledge of the statistics of the stimuli. The DCM rule is instead able to adapt to the case of unbalanced patterns without any modification, and achieves a much better performance, as can be seen in figure 5.

**Figure 6.**
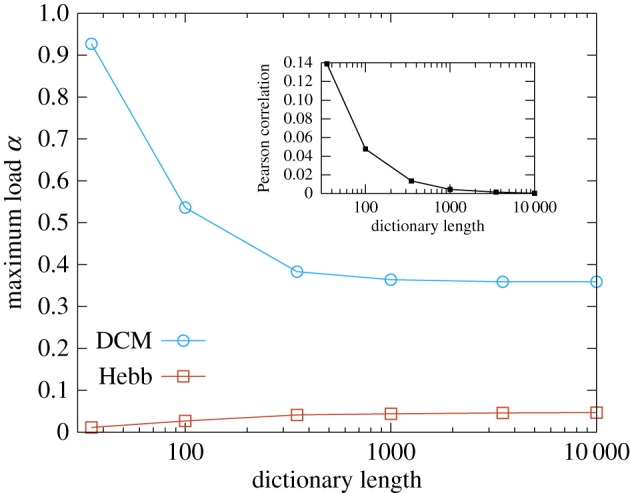
Maximum storage load as a function of the length of the dictionary of features. We study the critical capacity of the generalized Hebb rule (red curve) and the DCM rule (blue curve) when the patterns are generated as combinations of features, chosen from a dictionary of varying length *L*. In the inset, the mean Pearson correlation in a dataset of 200 patterns is shown as a function of the dictionary length. In the numerical experiments, every feature had a fixed sparsity of *f* = 0.1 and each pattern was obtained as a superposition of *F* = 6 features (see the electronic supplementary material, section *IV D*). The curves were obtained by averaging over 10 samples (error bars are smaller than the point size). (Online version in colour.)

A more realistic way of introducing pattern correlations can be studied in the 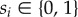 case, where it is possible to generate a set of patterns as combinations of sparse features drawn from a finite length *dictionary* (i.e. we pre-generate a set of sparse patterns—the dictionary of features 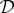
—and then generate each stimulus by taking a small random subset of 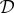 and superimposing the patterns within it; see the electronic supplementary material, section *IV D*). In the limit of an infinitely large dictionary, one produces uncorrelated patterns, but correlations set in as the length of the dictionary is reduced. In figure 6, we show how the DCM rule is able to take advantage of the decrease in the information content of the patterns as the total number of features is reduced.

**Figure 7.**
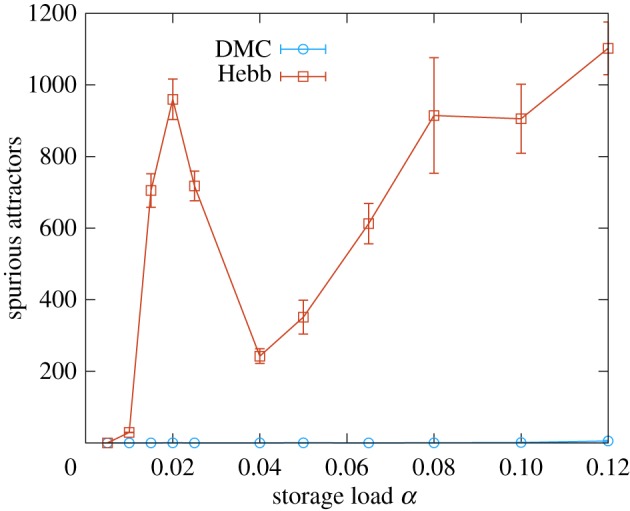
Number of spurious attractors for a network of *N* = 400 neurons. This figure shows the number of distinct spurious attractors found during 10000 independent random walks, of 200 time-steps, after a small number of patterns were learned by the network (see the electronic supplementary material, section *IV B*). The red curve represents the Hebb rule (the first peak is due to finite size effects). The blue curve shows the behaviour of the DCM rule. The curves were obtained by averaging over 10 samples (error bars are standard errors). (Online version in colour.)

Another drawback of the plain Hebb rule is the introduction of spurious memories while the desired patterns are embedded as attractors. These spurious states usually appear in overlapping regions of the basin of attraction of different stored memories, and are therefore referred to as mixture states [15]. As can be seen in figure 7, the problem of spurious attractors is almost completely avoided when the DCM rule is employed, since it is able to store the patterns more coherently and the basins of attraction are not likely to interfere with each other.

#### 2.2.2. One-shot learning

**Figure 8.**
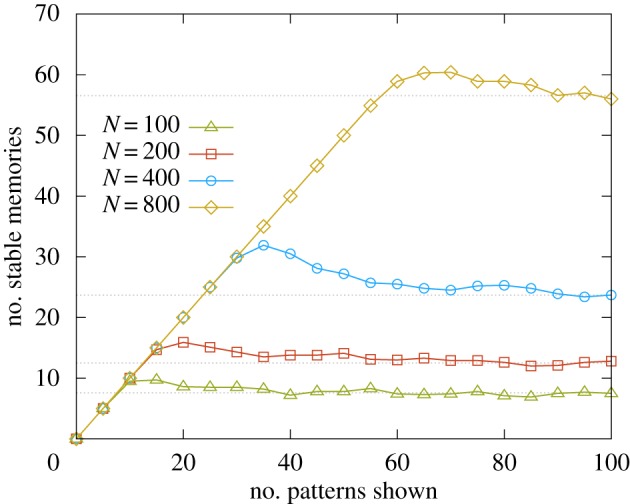
Scaling properties of the palimpsest capacity. In this figure, we show the results obtained when testing the DCM learning rule in the context of one-shot learning, for the case of 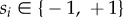 neurons. The full curves show the results for *N* = 100, 200, 400 and 800, illustrating the scaling properties of the palimpsest capacity. The dashed grey curves are extrapolated as the mean of the last three measurements. All the points are obtained by averaging over 10 samples. (Online version in colour.)

Finally, we also tested the DCM learning rule in a one-shot online setting: each pattern is presented to the network until it becomes a stable attractor and then is never seen again. In this scenario, the relevant measure of the performance is the so-called palimpsest capacity [47]: after an initial transient, the network is expected to enter a steady-state regime in which an old memory is lost every time a new one is learned. Our numerical results, obtained in the 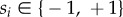 case (figure 8), show that—quite remarkably—by simply adding a weight regularization the DCM rule achieves an extensive palimpsest capacity, slightly above approximately 0.05*N*. This property was verified by a scaling analysis. Similar results can be obtained in the 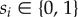 case only with the adaptive threshold regulatory scheme (see the electronic supplementary material, section *IV C*, for more details).

Another local learning rule that is known to perform well in an online setting was proposed by Storkey [48], and reads2.9
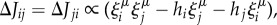
where 
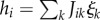
 are the local fields. The last two terms can penalize the weights when the memory is already stored (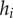 has the same sign of 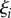) and the local field becomes excessively large, building a regularization mechanism directly into the learning rule. Limiting the growth of the synaptic weights is in fact necessary in order to avoid entering a spin glass phase, where all the memories are suddenly lost and learning can no longer take place [49]. However, Storkey’s rule fails when tested against our retrieval criterion in a finite temperature setting (we are setting 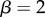 in the parallel Glauber dynamics). This not only shows that the DCM is able to embed attractors arbitrarily robustly (depending on the temperature considered during training), but also stresses the fact that the retrieval criterion that was employed throughout this paper is very strict compared to alternative definitions. For example, if we consider the criterion proposed in [48] the DCM rule palimpsest capacity is measured to be as high as approximately 0.3*N*.

### 2.3. Adding hidden neuronal states

When hidden neurons are introduced, the stochastic neural network turns into a rather general computational device, which can be framed as a parametric probabilistic model able to develop an internal representation of the statistics of external stimuli. This kind of neural network could recover a partially corrupted memory, as in an attractor neural network, but it could also be exploited as a generative model, able to produce new samples in accordance with the statistics inferred from the training data.

Even in the case with undirected symmetric synaptic couplings—the Boltzmann machine—the inference and learning problems become NP-hard, since the time required for the dynamics to reach thermal equilibrium is bound to grow exponentially with the network size [50]. A well-studied solution to these problems is to consider a simplified synaptic structure, in which the connections of the network are restricted to the ones between visible and hidden neurons, the so-called restricted Boltzmann machine (RBM) [51]. We will focus on the same rigid architecture.

The DCM learning rule can still be understood in a KL minimization framework. As before, in the infinite signal limit, we obtain a log-pseudo-likelihood optimization procedure, except that now the inference is from incomplete observations and an average over all the possible hidden neuronal states is required (see the electronic supplementary material, section *VI*). In this limit, the synaptic couplings are updated as2.10
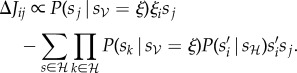


This equation is closely linked to the CD-*k* method, a heuristic algorithm for approximating the maximum-likelihood method for RBMs [21]. The first term in equation (2.10) requires sampling from the probability distribution of the hidden neuronal state induced by a clamping stimulus on the visible neurons, as in the positive phase of CD-*k*, while the second term can be estimated by implementing a Gibbs sampling chain starting from a visible state prepared in correspondence of the stimulus but subject to no external field, as in CD-*k*’s negative phase. This relationship could shed some light on the apparently surprising performance that can be obtained with CD, even when a very small number of Gibbs sampling steps *k* is chosen: this means that the partition function of the model is estimated very crudely, restricting sampling only to the mode induced by the seed of the Gibbs chain. This is in fact what the pseudo-likelihood method would require [24]. CD-*k*, however, is defined in the context of models with symmetric interactions and therefore does not apply to asymmetric kinetic models of the type considered throughout this work.

In the presence of hidden neurons, we can still apply the heuristic prescription described above (equation (2.6)), yielding a plasticity rule that matches time-delayed correlations, recorded during the network dynamics. In order to test numerically how a biologically plausible system could perform against a state-of-the-art learning method, we also derived the TAP mean-field equations [52] for approximating the steady-state distribution of the neural states and the time-delayed correlations (see the electronic supplementary material, section *V*, for their analytical derivation) in a sparse asymmetric network.

In the electronic supplementary material, section *VI*, we consider the problem of learning the statistics of a dataset of real-world images [53]. The performance of the DCM rule is assessed in the customary feature extraction, generative and classification tasks and compared with that of the TAP approach, on the same neural network architectures. While there is an obvious degradation in the learning performance, we also observe that the robustness of our learning model is still allowing the network to learn despite the presence of noise and strict detrimental biological constraints.

## 3. Discussion

In this work, we studied the problem of learning in general stochastic neural network models. Starting from a KL divergence minimization condition, we derived analytically a differential update rule closely related to the maximum pseudo-likelihood method, able to store an ensemble of patterns of neuronal activity, conveyed to the network in the form of external fields. With some slight modifications, we obtained a version of the rule that allowed us to introduce a number of important requirements for biological plausibility, concerning not only the network structure but the learning process as well. We further showed that all the needed information could be collected during the dynamics of the network by some kind of short-term memory mechanism, locally keeping track of correlations, and that the updates could be implemented by a comparator simply trying to maintain externally induced correlations by incrementing the synaptic weights.

Our DCM learning rule bears great resemblance with classical Hebb plasticity, in that synaptic modifications are driven only by the information about activity correlations locally available at the synapse. However, the DCM rule can be applied in a general framework where asymmetric synapses are allowed, at odds with the previous learning paradigms. Moreover, the rule relies on finite external signals, that are not able to quench the network dynamics completely. Apart from retaining a higher biological plausibility, this is one of the reasons why this rule can embed an extensive number of patterns while minimizing the pattern cross-talk, avoiding the creation of spurious memories. The stochastic network becomes capable of learning in a purely online context, including in the extreme limit of one-shot learning.

The differential form of the plasticity rule also allows for a good retrieval performance when the memories are correlated, both in the case of simply biased memories and in the case of patterns obtained as combinations of features. In the sparse case, we showed the robustness of the DCM rule to the introduction of the excitatory–inhibitory differentiation constraint (Dale’s principle), and proposed various inhibitory mechanisms which proved to be able to control the activity level of the network and to prevent the dynamics from reaching epileptic states.

Finally, we showed how the very same learning rule allows a more general network, in which hidden neurons are added, to perform well in feature extraction, generation and classification tasks, when dealing with real-world data. By means of comparison with a state-of-the-art method, we argue that, by implementing the proposed learning rule, a stochastic neural network obeying strong biological requirements could preserve great modelling potential. In particular, the similarities with Boltzmann machine learning [20,51] (see also below) suggest that the DCM rule may be a viable candidate for feature extraction and inference: for example, in experiments with patterns formed from combining features from a dictionary (as for those of figure 6), we may hope to recover the individual features as internal representations in the hidden part of the network. We performed preliminary experiments in this direction and the results are indeed promising. In this paper, however, our numerical analysis was limited to the well-studied case of directed visible-to-hidden synapses and digit recognition, and the exploration of hybrid and more general architectures and tasks is left for future work.

Future possible research directions include the generalization of this learning framework to continuous-time dynamics and more realistic spiking network models, and the problem of learning dynamical activation patterns instead of static ones. It must be noted that the idea of learning recurrent weight matrix in a network model by matching some measure of a driven system to that of an autonomous one is not new. The general strategy for stabilizing dynamical patterns has been rediscovered under several denominations in the broad context of reservoir computing and generally involves the matching of local *currents* [54–56], with notable examples both in the discrete time-step deterministic setting [57] and in spiking network models [58,59]. These models have the advantage of capturing the dynamical complexity of neural systems. We note that, on the other hand, they rely on some non-local learning strategies.

Our model also shares some similarities with the Equilibrium Propagation algorithm (EP) for energy-based models of [60], but with some crucial differences. The main similarity relies in the fact that the resulting update rule for the synaptic weights uses the difference between the correlations measured with the network in a weakly clamped state (using the EP terminology) and a free state. This is also reminiscent of the original algorithm for training Boltzmann machines proposed in [20]. The major difference in our model is the use of time-delayed correlations, which stems from the different approach used in our derivation and allows us to work in the general setting of asymmetric synaptic connections—indeed, the synaptic symmetry in the EP approach was regarded by the authors as its most unsatisfactory requirement from a biological perspective. Additional important differences arise from the overall setting and derivation: in the EP case, the context is supervised learning, the inputs are fully clamped and drive the network towards an equilibrium (in the free phase), after which the outputs are weakly clamped (the limit of vanishing clamping is considered) and the weights updated accordingly. In our case, the context is unsupervised learning, there is no preliminary equilibration step (the network is not reset between pattern presentations), and the external driving force is relatively weak but non-vanishing (it decreases to zero gradually as training progresses).

In [61], in the context of diluted neural networks, the authors used as a learning criterion the matching of *equal-time* correlations, still comparing a system driven by a finite field with a freely evolving one. In that case, however, the connections were assumed to be symmetric, and the correlations were estimated with the Belief Propagation algorithm. At odds with these approaches, we presented a formulation in terms of *delayed activity correlations* that, while requiring a time integration mechanism, is completely local, and is used to construct general excitatory–inhibitory asymmetric networks. Another attempt at devising a learning protocol with good performances and subject to basic biological constraints was presented in [34], exploiting the statistics of the inputs rather then the dynamical properties of the network. The resulting ‘three thresholds’ learning rule (3TLR) shares with the DCM rule most desirable features for a biological system, e.g. it can achieve near-optimal capacity even with correlated patterns. A detailed comparison of the performance of the two rules is technically and computationally demanding and unfortunately out of the scope of this work, but the 3TLR seems to require stronger driving external fields; furthermore, lowering the field results in an abrupt performance drop, while the DCM rule degrades gracefully (cf. figure 3).

## Supplementary Material

Supplementary Material

## Data accessibility

This article has no additional data.
