# Supplementary material for: From statistical inference to a differential learning rule for stochastic neural networks
Source: Interface Focus. 2018 Oct 19;8(6):20180033. doi: 10.1098/rsfs.2018.0033 (PMC6227809; doi:10.1098/rsfs.2018.0033)
Supplement: Supplementary Material [file rsfs20180033supp1.pdf]

From statistical inference to a differential learning rule for stochastic neural networks Supplementary Material

Luca Saglietti,[1, 2] Federica Gerace,[3, 2] Alessandro Ingrosso,[4] Carlo Baldassi,[5, 2, 6] and Riccardo Zecchina[5, 2, 7]

[1]*Microsoft Research New England, Cambridge (MA), USA*
[2]*Italian Institute for Genomic Medicine, Torino, Italy*
[3]*Politecnico di Torino, DISAT, Torino, Italy*
[4]*Center for Theoretical Neuroscience, Columbia University, New York, USA*
[5]*Bocconi Institute for Data Science and Analytics, Bocconi University, Milano, Italy*
[6]*Istituto Nazionale di Fisica Nucleare, Torino, Italy*
[7]*International Centre for Theoretical Physics, Trieste, Italy*

S1. TYPES OF NEURONS

In this work we considered two kinds of binary neurons, $s_i \in \{-1,+1\}$ and $s_i \in \{0,1\}$. The sigmoid-shaped function $\sigma(\cdot)$ which appears in eq. (1) of the main text takes two slightly different forms depending on the model, as a consequence of the different normalization term appearing in the two cases:

$$\sigma_{\pm 1}(s|h;\beta) = \frac{e^{\beta s h}}{e^{\beta h} + e^{-\beta h}} \tag{S1}$$

$$\sigma_{01}(s|h;\beta) = \frac{e^{\beta s h}}{1 + e^{\beta h}}. \tag{S2}$$

In the case of $s_i \in \{-1,+1\}$ neurons, we sampled each component of the patterns independently from a potentially biased probability distribution $P(\xi_i) = b\,\delta(\xi_i - 1) + (1-b)\,\delta(\xi_i + 1)$, with a bias parameter $0 < b < 1$. In most of our tests, however, we considered the unbiased case $b = 1/2$, except for those presented in fig. 5 of the main text. In this case, the local fields are naturally balanced around 0 and the thresholds θ_i can be eliminated.

In the case of $s_i \in \{0,1\}$ neurons, we sampled each component of the memories from the prior $P(\xi_i) = (1 - f_v)\,\delta(\xi_i) + f_v \delta(\xi_i - 1)$. Here f_v should also correspond to the network sparsity level, i.e. the average fraction of active neurons at a given time-step of the network dynamics, $f_v = \frac{1}{N}\sum_{i=1}^{N} s_i$. In this case, the thresholds θ_i are necessary to shift the distribution of the local fields around zero, and we used an inhibitory network to stabilize the overall activity (see the ‘Inhibitory Network models’ section below).

S2. ANALYTIC DERIVATION OF THE DCM LEARNING RULE

In this section we derive the equations for the DCM rule. For simplicity we will consider the case of $\beta = 1$. From a mathematical perspective, we ask our learning rule to reduce the Kullback-Leibler (KL) divergence between two different conditional probability distributions, $P(s'|s;\lambda_1)$ and $P(s'|s;\lambda_2)$, with $\lambda_2 < \lambda_1$, averaged over an initial state probability distribution $P(s)$. This quantity is given by:

$$\left\langle \mathrm{KL}\left(P(\cdot|s;\lambda_1)\,||P(\cdot|s;\lambda_2)\right)\right\rangle_P = \sum_s P(s) \sum_{s'} P(s'|s;\lambda_1) \log \frac{P(s'|s;\lambda_1)}{P(s'|s;\lambda_2)}. \tag{S3}$$

The conditional probability is defined as a sigmoid-shaped neural activation function (cf. eq. (1) of the main text)

$$P(s'|s;\lambda) = \prod_{i=1}^{N} \sigma\left(s_i'|h_i^\lambda\right), \tag{S4}$$

with local fields h_i^λ given by: $h_i^\lambda = h_i^{\mathrm{ext},\lambda} + \sum_{j\neq i} J_{ij}^\lambda s_j - \theta_i^\lambda$. Here we adopt the superscript λ to distinguish between the two networks, subject to different external field intensities λ.

Plugging the expression (S4) for the conditional probability into the definition (S3) of the KL divergence, we exploit the factorization property of the single neuron conditional probabilities, in order to isolate the i-th contribution and trace out all the others. Therefore, we get the final expression for the averaged KL divergence:

$$\left\langle \mathrm{KL}\left(P\left(\cdot|s;\lambda_1\right)||P\left(\cdot|s;\lambda_2\right)\right)\right\rangle_P = \sum_s P\left(s\right)\sum_i \sum_{s'_i} \sigma\left(s'_i|h_i^{\lambda_1}\right)\log\frac{\sigma\left(s'_i|h_i^{\lambda_1}\right)}{\sigma\left(s'_i|h_i^{\lambda_2}\right)} \tag{S5}$$

The next step is to minimize this quantity, by differentiating with respect to $J_{ik}^{\lambda_2}$ and $\theta_i^{\lambda_2}$, asking the second network to compensate for the decrease in the external field through an adaptation of its parameters. For both expressions of σ of eqs. (S1) and (S2), the following property holds:

$$\frac{1}{\beta}\frac{\partial}{\partial h}\log\sigma\left(s|h;\beta\right) = s - \left\langle s\right\rangle_h \tag{S6}$$

where here $\left\langle s\right\rangle_h = \sum_s s\,\sigma\left(s|h;\beta\right)$. This allows us to derive the following simple formulas for the derivatives with respect to the parameters, for both neuronal models:

$$\begin{aligned} -\frac{1}{\beta}\frac{\partial}{\partial J_{ik}^{\lambda_2}}\left\langle \mathrm{KL}\left(P\left(\cdot|s;\lambda_1\right)||P\left(\cdot|s;\lambda_2\right)\right)\right\rangle_P &= \sum_s P\left(s\right)\sum_{s'_i}\sigma\left(s'_i|h_i^{\lambda_1}\right)\left(s'_i - \left\langle s'_i\right\rangle\right)s_k \\ &= \left\langle s'_i s_k\right\rangle_{P,\lambda_1} - \left\langle s'_i s_k\right\rangle_{P,\lambda_2} \end{aligned} \tag{S7}$$

$$\begin{aligned} -\frac{1}{\beta}\frac{\partial}{\partial \theta_{i}^{\lambda_2}}\left\langle \mathrm{KL}\left(P\left(\cdot|s;\lambda_1\right)||P\left(\cdot|s;\lambda_2\right)\right)\right\rangle_P &= \sum_s P\left(s\right)\sum_{s'_i}\sigma\left(s'_i|h_i^{\lambda_1}\right)\left(s'_i - \left\langle s'_i\right\rangle\right) \\ &= -\left(\left\langle s'_i\right\rangle_{P,\lambda_1} - \left\langle s'_i\right\rangle_{P,\lambda_2}\right) \end{aligned} \tag{S8}$$

As mentioned above though, the second one is not actually used in the ± 1 model since we did not use the thresholds θ_i in that case.

A. Connection with maximum pseudo-likelihood method

In the fully visible case, the clamped probability distribution eq. (2) of the main text simply becomes $P_{\mathrm{clamp}}\left(s;\xi\right) = \prod_{i=1}^N \delta_{s_i,\xi_i}$, and the average KL divergence defined in eq. (S3) can be written explicitly as:

$$\begin{aligned} &\left\langle KL\left[P\left(\cdot|s;\lambda^{\mathrm{ext}}=\infty\right)||P\left(\cdot|s;\lambda^{\mathrm{ext}}=0\right)\right]\right\rangle_{P_{\mathrm{clamp}}(\xi)} = \\ &= -\sum_{i=1}^N \log P\left(s_i = \xi_i|\left\{s_j=\xi_j\right\}_{j\neq i};\lambda^{\mathrm{ext}}=0\right). \end{aligned} \tag{S9}$$

This expression can be recognized as one of the terms appearing in the so called log-pseudo-likelihood $\mathcal{L}\left(\left\{\xi^\mu\right\}|J_{ij},\theta;\beta\right) = \frac{1}{M}\sum_{\mu=1}^M\sum_{i=1}^N \log P\left(s_i=\xi_i^\mu|\left\{s_j=\xi_j^\mu\right\}_{j\neq i};\lambda^{ext}=0\right)$.

The pseudo-likelihood method provides a computationally inexpensive yet statistically consistent estimator [1] when the functional form of the joint probability distribution over the configurations is unknown, and is thus approximated in the factorized form $P\left(s=\xi^\mu\right) = \prod_i P\left(s_i=\xi_i^\mu|\left\{s_j=\xi_j^\mu\right\}_{j\neq i}\right)$. In the framework of learning, the minimization of eq. (S9) can be seen instead as a stability requirement for the memory ξ, as it progressively increases the probability that the stochastic dynamics remains fixed in the attractor state.

B. Connection with the perceptron rule

In the noise-free limit $\beta\to\infty$, where the state of the neuron s_i^{t+1} is deterministically obtained by taking the sign of the local incoming current, the pseudo-likelihood synaptic weight update would read:

$$\Delta J_{ij} = \begin{cases} 0 & \xi_i h_i \geq 0 \\ 2\eta\xi_i^\mu\xi_j^\mu & \xi_i h_i < 0 \end{cases}, \tag{S10}$$

which is the well-known perceptron rule. Indeed, since the next state of a neuron is conditionally dependent on the previous state of the other $N-1$ neurons, one can reinterpret the problem of learning a certain number of attractors as N independent perceptron learning problems. In a zero temperature setting, the incoming weights of a neuron i can be simply updated whenever its predicted state is misaligned with respect to the i-th component of the memory to be learned, $s_i^{t+1} \neq \xi_i$, by shifting its weights in the direction of the desired state and in parallel to the pattern itself. It is known that the perceptron rule saturates the theoretical Gardner bound $\alpha_c = 2$ for the critical memory capacity of a fully-visible neural network at zero noise [2].

Moreover, if we follow [3] and consider negative field intensities $\lambda_{\min} < 0$ (instead of $\lambda_{\min} = 0$ as in the pseudo-likelihood method), we obtain:

$$\Delta J_{ij} = \begin{cases} 0 & \xi_i h_i \geq |\lambda_{\min}| \\ 2\eta \xi_i^\mu \xi_j^\mu & \xi_i h_i < |\lambda_{\min}| \end{cases}. \tag{S11}$$

This is nothing but the perceptron rule with robustness parameter $|\lambda_{\min}|$, that forces the network to learn the memories so that they are attractive in a full sphere of such radius. However, any $\lambda_{\min} < 0$ will also cause the maximum capacity of the network to decrease [4].

S3. INHIBITORY NETWORK MODELS

We considered three different schemes that can reproduce the effect of an inhibitory network. In the first one, the inhibitory network is replaced by a global inhibitory unit connected to all the N excitatory neurons [5], which elastically drives the system towards the desired activity level through a feed-back signal. An alternative scheme can be obtained by introducing a soft "winner-takes-all" mechanism, effectively playing the role of a global inhibitory unit [6–16]. This mechanism is meant to model a continuous time scale phenomenon: the neurons with higher local activities could become active before the others and start to excite the inhibitory component of the network, whose feed-back signal is triggered when the correct fraction f_v of neurons is already active; this signal thus depresses all the local activities of the network, preventing the remaining neurons from activating. The last inhibitory scheme is based on the introduction of locally adaptive thresholds (from a biological point of view, this mechanism can be justified with the widely observed phenomenon of thresholds variability in the central nervous system [17]).

The aim of the inhibitory feedback is to maintain the excitatory network activity around a desired level, preventing epileptic (all-on) or completely switched off states in the $\{0, 1\}$ model. In the following, we provide more detailed explanations and some implementation details for each scheme.

A. The global inhibitory unit scheme

We consider a generalization of the global inhibitory unit scheme proposed in [5], for a purely excitatory stochastic neural network constituted by an ensemble of N neurons. Suppose that, within the entire neuronal population, we can distinguish G different groups of neurons, such that $N = \sum_{\alpha=1}^{G} N_\alpha$, with different sparsity levels. We introduce G global inhibitory units, whose task is to maintain the activity $S^\alpha = \sum_{i=1}^{N} s_i^\alpha$ of each population of neurons at the desired level $f^\alpha N_\alpha$. According to the global inhibitory unit scheme, each excitatory neuronal ensemble α receives a feed-back signal $\mathcal{I}^\alpha\left(\left\{f^\beta, S_\beta\right\}_{\beta=1}^{G}\right)$, which can be parametrized as:

$$\mathcal{I}^\alpha\left(\left\{f^\beta, S_\beta\right\}_{\beta=1}^{G}\right) = H_0^\alpha + \nu^{\alpha\alpha}\left(S_\alpha - f^\alpha N_\alpha\right) + \sum_{\beta \neq \alpha} \nu^{\alpha\beta}\left(S_\beta - f^\beta N_\beta\right). \tag{S12}$$

In this section we derive analytically an expression for both the global inhibition constant H_0^α and the parameters $\nu^{\alpha\beta}$ that control the elastic reaction to possible oscillations around the desired activities.

Assuming that the local fields h_i^α in population α are Gaussian distributed, the inhibitory units are required to correctly set the mean of the distribution around the mean threshold $T^\alpha = \langle \theta_i^\alpha \rangle$, so that the integral of the distribution above threshold contains exactly $f^\alpha N_\alpha$ local fields:

$$\langle h_i^\alpha \rangle = T^\alpha - H^{-1}\left(f^\alpha\right)\sigma_\alpha. \tag{S13}$$

Here $H^{-1}(x) = \sqrt{2}\mathrm{erfc}^{-1}(2x)$ represents an inverse error function, determining the proper shift to be applied, measured in units of the standard deviation of the distribution σ_α. The latter can be easily computed, giving:

$$\sigma_\alpha = \sqrt{\left(\sigma_J^{\alpha\alpha}\right)^2 \left(S^\alpha - f^\alpha\right) + \sum_{\beta\neq\alpha} \left(\sigma_J^{\alpha\beta}\right)^2 S^\beta}, \tag{S14}$$

where $\sigma_J^{\alpha\beta}$ stands for the standard deviation of the distribution of the synaptic couplings from population β to population α.

By summing and subtracting $\sum_\beta \left(\sigma_J^{\alpha\beta}\right)^2 f^\beta N_\beta$ in the square root, assuming small deviations of the activity of the network S^α from the desired activity level $f^\alpha N_\alpha$, we can expand σ_α obtaining:

$$\begin{aligned}\sigma_\alpha = &\sqrt{f^\alpha \left(N_\alpha - 1\right)\left(\sigma_J^{\alpha\alpha}\right)^2 + \sum_{\beta\neq\alpha} \left(\sigma_J^{\alpha\beta}\right)^2 f^\beta N_\beta} \times \\ &\times \left(1 + \frac{\left(\sigma_J^{\alpha\alpha}\right)^2 \left(S^\alpha - f^\alpha N_\alpha\right) + \sum_{\beta\neq\alpha}\left(\sigma_J^{\alpha\beta}\right)^2 \left(S^\beta - f^\beta N_\beta\right)}{2\left(f^\alpha \left(N_\alpha - 1\right)\left(\sigma_J^{\alpha\alpha}\right)^2 + \sum_{\beta\neq\alpha} \left(\sigma_J^{\alpha\beta}\right)^2 f^\beta N_\beta\right)}\right).\end{aligned} \tag{S15}$$

In the left hand side of eq. (S13), instead, each local field h_i^α is given by the sum of three different contributions, namely the external field, the recurrent input and the feed-back signal from the inhibitory unit:

$$\begin{aligned}\langle h_i^\alpha \rangle = \Big\langle & h_i^{\mathrm{ext},\alpha} + \sum_{j\neq i} J_{ij}^{\alpha\alpha} s_j^\alpha + \sum_{\beta\neq\alpha}\sum_j J_{ij}^{\alpha\beta} s_j^\beta - H_0^\alpha + \\ & -\nu^{\alpha\alpha}\left(S^\alpha - f^\alpha N^\alpha\right) - \sum_{\beta\neq\alpha} \nu^{\alpha\beta}\left(S^\beta - f^\beta N_\beta\right)\Big\rangle .\end{aligned} \tag{S16}$$

We can compute the average by summing and subtracting $\sum_\beta \overline{J^{\alpha\beta}} S^\beta$, obtaining:

$$\begin{aligned}\langle h_i^\alpha \rangle = &\overline{h^{\mathrm{ext},\alpha}} + \overline{J^{\alpha\alpha}}\left(S^\alpha - f^\alpha\right) - \sum_{\beta\neq\alpha} \overline{J^{\alpha\beta}} S^\beta - H_0^\alpha - \nu^{\alpha\alpha}\left(S^\alpha - f^\alpha N^\alpha\right) + \\ &- \sum_{\beta\neq\alpha} \nu^{\alpha\beta}\left(S^\beta - f^\beta N_\beta\right).\end{aligned} \tag{S17}$$

We therefore get an expression for the global inhibitory constant H_0^α and the parameters $\nu^{\alpha\alpha}$ and $\nu^{\alpha\beta}$ that satisfy eq. (S13):

$$\begin{aligned}H_0^\alpha = &\overline{h^{\mathrm{ext},\alpha}} + \left(N_\alpha - 1\right)\overline{J^{\alpha\alpha}} f^\alpha + \sum_{\beta\neq\alpha} N_\beta \overline{J^{\alpha\beta}} f^\beta + \\ &+ H^{-1}\left(f^\alpha\right)\sqrt{f^\alpha\left(N_\alpha - 1\right)\left(\sigma_J^{\alpha\alpha}\right)^2 + \sum_{\beta\neq\alpha}\left(\sigma_J^{\alpha\beta}\right)^2 f^\beta N_\beta} - T^\alpha\end{aligned} \tag{S18}$$

$$\nu^{\alpha\alpha} = \overline{J^{\alpha\alpha}} + \frac{H^{-1}\left(f^\alpha\right)\left(\sigma_J^{\alpha\alpha}\right)^2}{2\sqrt{f^\alpha\left(N_\alpha - 1\right)\left(\sigma_J^{\alpha\alpha}\right)^2 + \sum_{\beta\neq\alpha}\left(\sigma_J^{\alpha\beta}\right)^2 f^\beta N_\beta}} \tag{S19}$$

$$\nu^{\alpha\beta} = \overline{J^{\alpha\beta}} + \frac{H^{-1}\left(f^\alpha\right)\left(\sigma_J^{\alpha\beta}\right)^2}{2\sqrt{f^\alpha\left(N_\alpha - 1\right)\left(\sigma_J^{\alpha\alpha}\right)^2 + \sum_{\beta\neq\alpha}\left(\sigma_J^{\alpha\beta}\right)^2 f^\beta N_\beta}}. \tag{S20}$$

Notice that a contribution to the global inhibitory constant H_0^α arises from the mean external field $\overline{h^{\mathrm{ext},\alpha}} = \lambda^{\mathrm{ext}} f^\alpha$, so that neurons that do not receive an excitatory external stimulus are effectively depressed. Since the adaptation of the synaptic couplings according to the plasticity rule is considered to be adiabatic, the means and the standard deviations required for setting a correct inhibition are affected only over longer time scales and need not be updated instantaneously.

The scheme described here can be easily specialized to the simple cases of fully visible or visible-to-hidden restricted connectivity, which have been analyzed in detail in this work.

B. Soft "winner takes all" mechanism

This inhibitory scheme can be easily implemented in the synchronous dynamics considered in this work: before the new neuronal state gets extracted (eq. (1) of the main text), the local activities are first sorted with respect to their magnitude, then a global inhibitory input is added, whose value is set just below the activation of the (fN)-th highest excited neuron. This procedure guarantees a fine-tuned control on the sparsity level f of the network. When the network is composed of a number G of different groups of neurons, each with a different sparsity level, the sorting operation is done inside each group. Some theoretical results show that neurons with adaptive threshold perform better than those with a constant threshold in presence of highly correlated stimuli [18]: we confirm these observations, since we have seen that this scheme is the best one in the one-shot learning task.

C. The adaptive thresholds regulatory scheme

The $s_i \in \{0,1\}$ case can be mapped exactly on the $s_i \in \{-1,+1\}$ case, but this operation requires the thresholds to dynamically adapt to any change in the synaptic couplings.

In order to obtain the correct mapping one can consider the conditional probabilities of the two models, and look for a transformation of the neural variables and of the parameters which allows to move between the two scenarios. After inserting the simple change of variables $s_i \to s_i' = \frac{(s_i+1)}{2}$ in the expression for the local activities in the $s_i \in \{-1,+1\}$ model (note that in this section the s' notation is *not* used to denote the next step of the dynamics), we get the matching equation:

$$h_i' = 2\left(h_i^{\text{ext}} + \sum_j J_{ij}\left(2s_i' - 1\right)\right) = h_i^{\text{ext}\,\prime} + \sum_j J_{ij}' s_i' - \theta_i'. \tag{S21}$$

which is satisfied by posing $h_i^{\text{ext}\,\prime} = 2h_i^{\text{ext}}$, $J_{ij}' = 4J_{ij}$ and $\theta_i' = 2\sum_j J_{ij} = \frac{1}{2}\sum_j J_{ij}'$ in the case of $s_i \in \{0,1\}$ neurons.

By looking at the case with $f_v = 0.5$ sparsity, this mapping suggests that it is possible to set the thresholds in correspondence of the average value of the incoming excitatory stimuli received by each neuron:

$$\theta_i = \left\langle \sum_{j\neq i} J_{ij} s_j^t \right\rangle_t = f_v \sum_{j\neq i} J_{ij}. \tag{S22}$$

This definition properly matches the one obtained from the exact mapping just in the case of $f_v = 0.5$. However, this choice was found to allow an extensive capacity in the on-line learning regime for the $s_i \in \{0,1\}$ neuronal state variables even with different sparsity levels. Having set the thresholds in such a way, one gets a slightly different form for the local activations $h_i = h_i^{\text{ext}} + \sum_{j\neq i} J_{ij}\left(s_j^t - f_v\right)$ and the learning rule eq. (S7) changes to:

$$\Delta J_{ij} \propto \left(\left\langle s_i^{t+1}\left(s_j^t - f_v\right)\right\rangle_{t,\lambda_1} - \left\langle s_i^{t+1}\left(s_j^t - f_v\right)\right\rangle_{t,\lambda_2}\right). \tag{S23}$$

S4. SIMULATION: IMPLEMENTATION DETAILS

We provide here detailed description of the learning algorithm in its heuristic version, described at the end of 'The Model' section, with the update rule eq. (6) of the main text. The learning protocol consists of an iterative optimization procedure where the parameters J and θ are incrementally updated. Throughout this work we initialized the weights J uniformly at random; for the ± 1 models, they were sampled from the interval $\left[-\frac{1}{\sqrt{N}}, \frac{1}{\sqrt{N}}\right]$, while for the $0/1$ models they were sampled from the interval $\left[0, \frac{1}{\sqrt{N}}\right]$. The thresholds θ were set to 0 in the ± 1 model, and initialized all to the same value in the $0/1$ model (the precise value is essentially irrelevant because of the effect of the inhibitory network; we used 0.35 in our simulations).

Every pattern ξ^μ is presented in the form of an external field $h^{\text{ext}} = \lambda^{\text{ext}}\xi^\mu$, where the signal intensity is initialized at a fixed value $\lambda_{\max}$ and then progressively decreased to zero in steps of $\Delta\lambda$. Before the learning process starts, we let the network evolve towards a state correlated with the pattern by waiting for a few iterations T_{init} of the dynamics

while the external field set to its maximum. Then, the learning process starts in the "positive" phase, registering the correlations in a time window of T steps at an initial value of the external field λ, and subsequently at a value lowered by $\Delta\lambda$, in the "negative" phase. The parameters are updated with a fixed learning rate η, as in eq. (6) of the main text.

This procedure is repeated until the external field reaches zero. The length of the time windows T has to be chosen in such a way that the state reached at the end of each averaging procedure is still in nearly the same region around the pattern, otherwise another initialization phase would be needed. In our experiments, we found that a good performance is achieved when T ranges from ~ 3 to ~ 25, provided the learning rate is lowered when the average is taken over very few iterations. This shows that a network implementing the DCM plasticity rule is able to learn even in the presence of an extremely low signal-to-noise ratio.

The relevant computation can be parallelized, since all the quantities involved (both in the dynamics and in the learning process) are local with respect to the synapses and the neurons. A simple pseudo-code implementation scheme for the learning protocol can be found in algorithm 1.

The learning rate is constant in time and was arbitrarily set to $\eta = 0.01$ in our simulations.

Input: parameters: η, *cycles*, λ_{max}, $\Delta\lambda$, T, T_{init}
Initialize J randomly $\sim U\left(\frac{-1}{\sqrt{N}}, \frac{1}{\sqrt{N}}\right)$;
for *cycle* $= 1$ **to** *cycles* **do**
  for μ *in random permutation of* $[1:p]$ **do**
    Set the external field on the visible neurons to an intensity λ_{max};
    Run the network for T_{init} steps;
    while $\lambda > 0$ **do**
      Estimate $\langle s_i^{t+1} s_j^t \rangle_\lambda$ for T steps;
      Estimate $\langle s_i^{t+1} s_j^t \rangle_{\lambda-\Delta\lambda}$ for T steps;
      $J_{ij} \leftarrow J_{ij} + \eta \left[\langle s_i^{t+1} s_j^t \rangle_\lambda - \langle s_i^{t+1} s_j^t \rangle_{\lambda-\Delta\lambda} \right]$;
      $\lambda \leftarrow \lambda - \Delta\lambda$;
    end
  end
  if *all patterns are learned* **then**
    BREAK;
  end
end

Algorithm 1: Pseudo-code implementation scheme for the DCM learning protocol (fig. 1 of the main text). For simplicity, we report the scheme used for ± 1 network models.

A. Measuring the width of the basins of attraction

We introduced an operative measure of the basin size, relating it to the level of corruption of the memories before the retrieval: a set of $M = \alpha N$ patterns is considered to be successfully stored at a noise level χ if, initializing the dynamics in a state where a fraction χ of the pattern is randomly corrupted, the retrieval rate for each pattern is at least 90% (as estimated from 100 separate trials per pattern) after at most 1000 learning cycles (250 in the simulations with finite fields). A successful retrieval is measured when, in absence of external input, the network evolves towards a neuronal state with an overlap ≥ 0.99 with the learned pattern in at most 50 steps of the dynamics.

B. Spurious attractors

In the numerical experiments for fig. 7 of the main text, the storage load α was chosen to be sufficiently small, such that both the DCM rule and the Hebb rule are able to learn stable attractors. The presence of spurious attractors was detected as follows: the network state was initialized at random, and was then allowed to evolve freely for 200 time steps. After this initialization period, the magnetization was recorded for a few iterations and compared with the stored attractors. If the modulus of the overlap with any one of them was > 0.95, the state was considered to have reached a known attractor. Otherwise, the magnetization was recorded for some more iterations, in order to check if a stable state was reached, and if this condition occurred the magnetization was clipped to ± 1 and a new spurious attractor was counted. In the following random restart, this attractor was inserted in the list of known attractors.

Of course, this procedure only provides an estimate of the number of distinct spurious attractors introduced by the learning rule, but sufficient to highlight a large, qualitative difference in the behavior of DCM compared to the Hebb rule.

The first peak in fig. 7 of the main text, in the Hebb's curve plot, is due to finite size effects: the number of spurious states is expected to grow at least exponentially in a sub-extensive regime $M \ll N$. In the extensive regime, mixtures of odd number of memories can still be observed, but as the storage load α is increased the mixture states composed of larger number of patterns are expected to disappear, and the growth in the number of spurious attractors is no longer exponential [19].

C. One-shot tests and palimpsest regime

In the one-shot simulations every pattern is seen by the network only once, and its memory will eventually be overwritten by the new ones. The goal is to reach a steady state regime in which, at each new presentation, the last M learned memories maintain the required stability. This storage load is called the palimpsest capacity.

In order to reach the maximal capacity, the parameters have to be fine tuned so that the learning process for each memory is slow and the most recently learned ones are minimally perturbed: one has to ensure that in the freely-evolving dynamics, i.e. the last time window during the external field pulse, the neuronal state does not escape the basin of attraction of the new memory and enters a previously learned one, causing the loss of existing memories. In the simulations presented in fig. 8 of the main text we addressed this problem rather drastically by simply removing this last window, which only resulted in a slight improvement in the palimpsest capacity.

We also set $\eta = 0.01$, $\lambda_{max} = 4$, $\Delta\lambda = 1$ and the length of the time windows was chosen to be slightly faster then in the other tests, $T = 10$. In this setting the number of presentations of the same pattern, i.e. the number of external field pulses, required for reaching its desired stability is around ~ 1000. This number would grow in time, because of the increase in the average connectivity of the network as new memories are added, a problem that can be overcome with the introduction of a synaptic weight $L2$-regularization.

In the case of $s_i \in \{0, 1\}$ neurons, we surprisingly lose the property of an extensive palimpsest capacity. The problem seems to be related to the need for a substantial shift in the threshold, that would allow a wide basin of attraction for a new pattern, as expressed by eq. (6) of the main text. This modification seems to strongly affect the network dynamics also when it hovers around a different, previously learned memory, introducing a disruptive effect in the palimpsest regime. In the normal learning task, instead, the thresholds are eventually set to a level which is compatible with all the patterns, since the learning protocol can cycle through the pattern set many times. The only way we found to obtain a good performance in the one-shot learning task for $s_i \in \{0, 1\}$ neurons with our model is to introduce an adaptive threshold regulatory scheme, stemming from a direct mapping to the $s_i \in \{-1, +1\}$ case.

D. Generation of correlated patterns

In the case of $s_i \in \{-1, +1\}$ neurons, we only introduced correlations in the form of a bias in the generation of the patterns, see section 'Types of neurons' above. Note that, in the biased case $b \neq 1/2$, it is known that the naive Hebb rule $J_{ij} = \frac{1}{M} \sum_\mu \xi_i^\mu \xi_j^\mu$ has to be generalized to $J_{ij} = \frac{1}{M} \sum_\mu (\xi_i^\mu - 2b + 1) \left(\xi_j^\mu - 2b + 1\right)$.

In the $s_i \in \{0, 1\}$ case, instead, we also generated correlated patterns as combinations of sparse features ϕ^ν, with $P\left(\phi_i^\nu\right) = f\,\delta\left(\phi_i^\nu - 1\right) + (1 - f)\,\delta\left(\phi_i^\nu\right)$, chosen from a finite length dictionary $\mathcal{D} = \{\phi^\nu\}_{\nu=1}^{L}$. Every pattern contains a fixed number of features, F, and its components can be written as: $\xi_i^\mu = \Theta\left(\sum_\nu c_\nu^\mu \phi_i^\nu\right)$, with $c_\nu^\mu \in \{0, 1\}$ determining whether the feature ν appears in pattern μ, and $\Theta(\cdot)$ is the Heaviside theta function, $\Theta(x) = 1$ if $x > 0$ and $\Theta(x) = 0$ otherwise.

S5. TAP APPROXIMATION IN ASYMMETRIC SPARSE MODELS

In the heuristic version of DCM, the time-delayed correlations of a network subject to varying external field intensities are needed in order to update the model parameters. In our approach, we employed a Monte Carlo scheme – which relies solely on the network dynamics – for their evaluation, as a means to fulfill some basic biological constraints. A better approximation though can be achieved with the so-called TAP approach, consisting in a second order expansion around a mean field limit, which can provide an estimation for the marginal probabilities of the neuronal state variables. The related magnetizations can then be used to compute approximate values for the pairwise correlations.

In what follows, we will apply the same procedure proposed in ref. [20] for the $s_i \in \{-1, +1\}$ case and the sequential Glauber dynamics, to the $s_i \in \{0, 1\}$ and the synchronized dynamics case. Since we are dealing with an

asymmetric model, where the form of the joint probability distribution $P(s|\theta,J)$ is unknown, we have to assume a weakly interacting regime, with small $\mathcal{O}\left(1/\sqrt{N}\right)$ couplings J, and in addition to be close to a mean field model with a factorized distribution:

$$P^{\mathrm{MF}}\left(s|\theta^{\mathrm{MF}}\right)=\prod_{a=1}^{N}\frac{\exp\left(\theta_a^{\mathrm{MF}}s_a\right)}{1+\exp\left(\theta_a^{\mathrm{MF}}\right)}. \tag{S24}$$

We introduce the parametrization $\theta_a^{\mathrm{MF}}=\theta_a-d\theta_a$ where $d\theta_a$ is small and θ^{MF} are the parameters of the mean field model, which can be found by minimizing the KL divergence:

$$KL\left[P||P^{\mathrm{MF}}\right]=\sum_s P(s|\theta,J)\log\left(\frac{P(s|\theta,J)}{P^{\mathrm{MF}}\left(s|\theta^{\mathrm{MF}}\right)}\right). \tag{S25}$$

The TAP approximation is obtained by performing a Taylor expansion of the magnetizations $m_a=\sum_s P(s_a)\,s_a$ in the small parameters J_{jk} and $d\theta_i$ and applying the matching condition $m_a-m_a^{\mathrm{MF}}=0$ for all $a\in\{1,\ldots,N\}$ up to second order:

$$\begin{aligned}0=m_a-m_a^{\mathrm{MF}}\approx&\sum_i\left.\frac{\partial m_a}{\partial\theta_i}\right|_{\mathrm{MF}}d\theta_i+\sum_{i<j}\left.\frac{\partial m_a}{\partial J_{ij}}\right|_{\mathrm{MF}}dJ_{ij}+\\&+\sum_{ij}\left.\frac{\partial^2 m_a}{\partial\theta_i\partial\theta_j}\right|_{\mathrm{MF}}d\theta_i d\theta_j+\sum_{i<j}\sum_{k<l}\left.\frac{\partial^2 m_a}{\partial J_{ij}\partial J_{kl}}\right|_{\mathrm{MF}}dJ_{ij}dJ_{kl}+\\&+2\sum_{i<j}\sum_k\left.\frac{\partial^2 m_a}{\partial J_{ij}\partial\theta_k}\right|_{\mathrm{MF}}dJ_{ij}d\theta_k\end{aligned} \tag{S26}$$

After some calculations, the following derivatives, evaluated in correspondence of the mean field probability distribution, are obtained:

$$\left.\frac{\partial m_a}{\partial\theta_i}\right|_{\mathrm{MF}}=m_a(1-m_a)\,\delta_{ai} \tag{S27}$$

$$\left.\frac{\partial m_a}{\partial J_{ij}}\right|_{\mathrm{MF}}=m_j m_a(1-m_a)\,\delta_{ai} \tag{S28}$$

$$\left.\frac{\partial^2 m_a}{\partial\theta_i\partial\theta_j}\right|_{\mathrm{MF}}=\left(m_a(1-m_a)^2-(m_a)^2(1-m_a)\right)\delta_{ai}\delta_{aj} \tag{S29}$$

$$\begin{aligned}\left.\frac{\partial^2 m_a}{\partial J_{ij}\partial\theta_k}\right|_{\mathrm{MF}}=&\,m_j m_a(1-m_j)(1-m_a)\,\delta_{ai}\delta_{jk}+\\&+m_j\left[m_a(1-m_a)^2-(m_a)^2(1-m_a)\right]\delta_{ai}\delta_{ak}\end{aligned} \tag{S30}$$

$$\begin{aligned}\left.\frac{\partial^2 m_a}{\partial J_{ij}\partial J_{kl}}\right|_{\mathrm{MF}}=&\,m_j m_l(1-m_l)\,m_a(1-m_a)\,\delta_{li}\delta_{ak}+\\&+m_l m_j(1-m_j)\,m_a(1-m_a)\,\delta_{jk}\delta_{ai}+\\&+\langle s_j s_l\rangle_{\mathrm{MF}}\left(m_a(1-m_a)^2-(m_a)^2(1-m_a)\right)\delta_{ai}\delta_{ak}.\end{aligned} \tag{S31}$$

Using the identity $\langle s_j s_l\rangle_{\mathrm{MF}}=\delta_{jl}m_j+(1-\delta_{jl})\,m_j m_l$ and neglecting higher orders up to $\mathcal{O}\left(d\Theta^2\right)$, the moment matching condition reads:

$$\theta_a^{\mathrm{MF}}=\theta_a+\sum_j m_j J_{aj}+\frac{1}{2}(1-2m_a)\sum_j\left(m_j(1-m_j)\right)J_{aj}^2. \tag{S32}$$

This leads to the TAP equations for the single neuron marginal probabilities when the sigmoid activation function is applied. The fixed point of these equations can be found by recursion, with a *crucial caveat*, namely that during the

iterative procedure the time indices of the magnetization appearing in the Onsager reaction term have to be chosen carefully, according to:

$$m_i^{t+1} = \mathrm{sigm}\left(\theta_i + \sum_j m_j^t J_{ij} - \left(m_i^{t-1} - \frac{1}{2}\right)\sum_j \left(m_j^t\left(1 - m_j^t\right)\right) J_{ij}^2\right) \tag{S33}$$

where $\mathrm{sigm}\left(x\right) = \left(1 + e^{-x}\right)^{-1}$ is the sigmoid function. Note that in the model presented in the main text the constant field θ_i is further decomposed into the effect of an external field and of a negative threshold $\theta_i \to \lambda^{\mathrm{ext}}\left(\xi_i - \frac{1}{2}\right) - \tilde{\theta}_i$.

Once the magnetizations are estimated, one can calculate the time-delayed correlations in the same TAP approximation. The dependence of these correlations on the magnetizations can be derived starting from:

$$\langle s_i' s_j \rangle = \sum_s P\left(s\right) s_j \sum_{s_i'} P\left(s_i' | s\right) s_i'. \tag{S34}$$

After expanding the sum over s_i', one simply obtains: $\langle s_i' s_j \rangle = \langle \mathrm{sigm}\left(h_i\right) s_j \rangle$. In order to simplify some of the following derivations, we first consider the Taylor expansion of the connected time-delayed correlations:

$$\chi_{ij}^D = \langle s_i' s_j \rangle - m_i m_j = \langle s_i \left(\mathrm{sigm}\left(h_j\right) - m_j\right) \rangle . \tag{S35}$$

In order to find an expression up to second order in $d\Theta$, we need the following derivatives:

$$\left.\frac{\partial \chi_{ba}^D}{\partial \theta_i}\right|_{\mathrm{MF}} = 0 \tag{S36}$$

$$\left.\frac{\partial \chi_{ba}^D}{\partial J_{ij}}\right|_{\mathrm{MF}} = m_b m_a \left(1 - m_b\right)\left(1 - m_j\right) \delta_{aj} \delta_{bi} \tag{S37}$$

$$\left.\frac{\partial^2 \chi_{ba}^D}{\partial \theta_i \partial \theta_j}\right|_{\mathrm{MF}} = 0 \tag{S38}$$

$$\left.\frac{\partial^2 \chi_{ba}^D}{\partial J_{ij} \partial \theta_k}\right|_{\mathrm{MF}} = m_a m_b \left(1 - m_a\right)\left(1 - m_b\right)\left(1 - 2m_b\right) \delta_{aj} \delta_{bk} \delta_{bi} \tag{S39}$$

$$\left.\frac{\partial^2 \chi_{ba}^D}{\partial J_{ij} \partial J_{kl}}\right|_{\mathrm{MF}} = m_a m_b \left(1 - m_b\right)\left(1 - 2m_b\right) \delta_{bk} \delta_{bi} \times \tag{S40}$$

$$\times \left(\delta_{aj}\delta_{al} + \left(1 - \delta_{aj}\right) \delta_{al} m_j \left(1 - m_a\right)\right). \tag{S41}$$

Using the following relation:

$$\begin{aligned}\langle s_a s_j s_l \rangle_{\mathrm{MF}} = {}& \delta_{aj}\left(\delta_{al} m_a + \left(1 - \delta_{al}\right) m_a m_l\right) + \\ & + \left(1 - \delta_{aj}\right)\left(\delta_{al} m_a m_j + \left(1 - \delta_{al}\right) m_a \langle s_j s_l \rangle_{\mathrm{MF}}\right),\end{aligned} \tag{S42}$$

we obtain the expression for the Taylor expansion up to second order:

$$\chi_{ij}^D = \left(m_i\left(1 - m_i\right)\right)\left(m_j\left(1 - m_j\right)\right)\left(J_{ij} + \frac{1}{2}\left(2m_i - 1\right)\left(2m_j - 1\right)\left(J_{ij}\right)^2\right), \tag{S43}$$

and therefore the final expression for the time-delayed correlations reads:

$$\begin{aligned}\langle s_i' s_j \rangle = {}& \left(m_i\left(1 - m_i\right)\right)\left(m_j\left(1 - m_j\right)\right) \times \\ & \times \left(J_{ij} + \frac{1}{2}\left(2m_i - 1\right)\left(2m_j - 1\right)\left(J_{ij}\right)^2\right) + m_i m_j.\end{aligned} \tag{S44}$$

S6. VISIBLE-TO-HIDDEN DIRECTED SYNAPSES

In the case of an architecture restricted to visible-to-hidden directed connections, the network can be seen as a bipartite graph. At any given time the state of a neuron in one of the two subsets is conditionally dependent only on the state of the complementary subset of neurons at the previous time:

$$P\left(s_i', i \in \mathcal{V} | s\right) = P\left(s_i', i \in \mathcal{V} | s_{\mathcal{H}}\right) \tag{S45}$$

$$P\left(s_i', i \in \mathcal{H} | s\right) = P\left(s_i', i \in \mathcal{H} | s_{\mathcal{V}}\right). \tag{S46}$$

Because of this property the joint conditional probability $P\left(s_{\mathcal{H}} | s_{\mathcal{V}}\right)$ can be factorized, and the clamped probability distribution can be written explicitly:

$$P_{\text{clamp}}\left(s; \xi\right) = \prod_{i \in \mathcal{V}} \delta_{s_i, \xi_i} \prod_{j \in \mathcal{H}} P\left(s_j | s_{\mathcal{V}} = \xi\right). \tag{S47}$$

The learning rule can be derived from the minimization of the KL divergence between the conditional probabilities obtained when the external field intensity is $\lambda^{ext} = \infty$ and $\lambda^{ext} = 0$, averaged over the clamped probability distribution. By differentiating the KL with respect to a hidden to visible synaptic coupling J_{ij} with $i \in \mathcal{V}$, $j \in H$, we get the following update rule:

$$\Delta J_{ij} \propto P\left(s_j | s_{\mathcal{V}} = \xi\right) \xi_i s_j - \sum_{s \in \mathcal{H}} \prod_{k \in \mathcal{H}} P\left(s_k | s_{\mathcal{V}} = \xi\right) P\left(s_i' | s_{\mathcal{H}}\right) s_i' s_j. \tag{S48}$$

As in the case of fully visible networks, the same increment would be obtained if an on-line optimization of the pseudo-likelihood of the model was instead implemented, except that now its estimation implies an average over all the possible hidden neuronal states:

$$\mathcal{L}\left(\{\xi^\mu\} | J_{ij}, \theta; \beta\right) = \frac{1}{M} \sum_{\mu=1}^{M} \sum_{i \in \mathcal{V}} \log \left(\sum_{s_j \in \mathcal{H}} \prod_{k \in \mathcal{H}} P\left(s_k | s_{\mathcal{V}} = \xi\right) P\left(s_i' = \xi_i^\mu | s_{\mathcal{H}}; \lambda^{ext} = 0\right) \right). \tag{S49}$$

A. MNIST Simulations

Instead of trying to construct an artificial stimulus ensemble, we use the MNIST database benchmark [21], which consists of $7 \cdot 10^4$ 28×28 grayscale images representing hand-written digits in 10 classes from 0 to 9. Images are sparse, with an average luminosity of $\overline{f_\xi} = 0.13066$ and every component ranging in the interval $\xi_i^\mu \in [0, 1]$. It is rather natural to consider each pattern as an array of probabilities of finding the corresponding neurons in the active state: we therefore consider a stochastic network of $s_i \in \{0, 1\}$ neurons, whose visible component will be successively subject to an external field corresponding to each one of the images, as before multiplied by a field intensity λ^{ext}. We hold out the last 10^4 images as a test set for the generalization performance, and employ the first $6 \cdot 10^4$ images to learn the statistics of the data.

We consider an architecture with $|\mathcal{V}| = 784 + 10$ visible neurons, plus $|\mathcal{H}| = 1000$ hidden neurons to guarantee a high representational capacity. The additional 10 visible neurons, one for each digit, can allow the network to learn input-output correlations: these neurons received a supervised input indicating the correct label of the image during the learning phase [22], and were present in all the simulations described in the following; however, they are exploited only for the classification task, being unessential for the usual generative tasks.

In order to point out how the DCM is able to deal with all the biological constraints we are considering in this work, we offer the direct comparison between two different learning models. In the first numerical experiment, which serves as a benchmark reference, the network was trained in the infinite signal limit ($\lambda_{\max} \sim 50$ is sufficient) corresponding to the pseudo-likelihood method, with unconstrained synapses, no inhibitory mechanism and using the time-delayed correlations obtained in the TAP approximation. In the second numerical experiment, meant to test the DCM rule

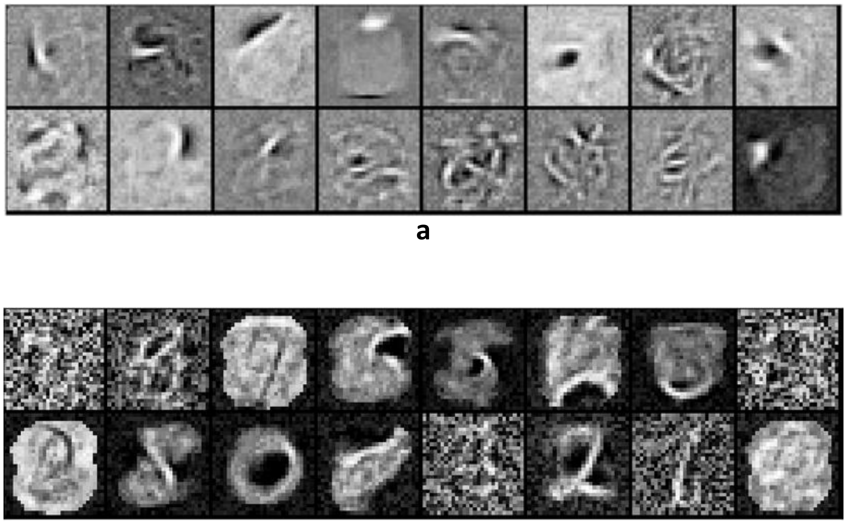

Figure S1. *Receptive fields of the hidden neurons.* In this figure we show some of the receptive fields of the neurons belonging to the hidden subset, in the two proposed experiments. *a.* Receptive fields learned in the first experiment, where the TAP approximation was employed in the clamped limit with no inhibition. *b.* Receptive fields learned in the second experiments, where the correlation were registered during the time evolution of the network, and where finite time-dependent fields, constrained synapses and the "winner-takes-all" inhibition scheme where considered.

in a more biologically plausible, we studied a purely excitatory network and implemented the soft "winner-takes-all" inhibitory scheme, fixing an average hidden activity of $f_h = 0.2$. The network was required to learn from finite external fields ($\lambda_{\max} = 3$, $\Delta\lambda = 3/2$ and $\lambda_{\min} = 0$) and to estimate the correlations simply through its own dynamics (specifically, we considered $T = 15$), as described in sec. 1. In both experiments the networks cycled 2 times through the $6 \cdot 10^4$ training images of the MNIST dataset.

In the second experiment setting, a very high level of noise can become extremely detrimental: with large hidden layers the network is often prone to falling into a completely symmetric state, with very poor performance. One would instead want to exploit the initial randomness in the synaptic couplings as a tool for breaking this otherwise problematic symmetry between the hidden neurons. This can be either achieved by choosing a lower temperature $\beta = 30$ (we choose this setting, to be compared with $\beta = 2$ in the first experiment) or by rescaling the initial random configuration of the synaptic couplings.

Receptive fields

A first comparison of the learning performance in the two cases is attained by visualizing the receptive fields of the hidden neurons, which can show how each different hidden unit specializes in the detection of a unique feature of the pattern set learned by the neural network. The receptive fields of the hidden neurons are represented by the synapses J_{ij} with a fixed $i \in \mathcal{H}$ and j running through the visible indices $\mathcal{V}$. These arrays can be reorganized as a 28×28 grayscale images as well, after renormalizing each component in the interval $[0, 1]$: the obtained image represents, for any hidden unit, its optimal stimulus. A sample is shown in fig. S1.

It is apparent that most of the hidden units develop interesting internal representations which can be interpreted as simple detectors for edges of parts of single digits. Both experiments also show the presence of a small fraction of extremely noisy features (that usually become irrelevant since the threshold of the corresponding neuron raises in order to inhibit its activation during the dynamics).

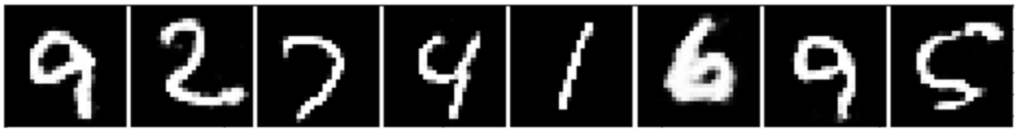

a

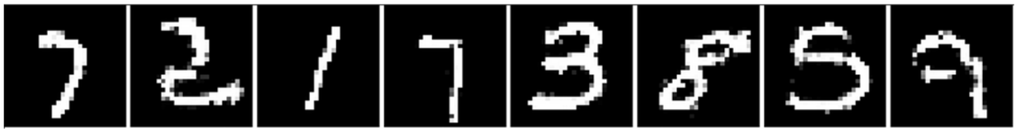

b

Figure S2. *Generation of samples.* The two series of plots show the probability of obtaining an active state for the visible neurons after 100 time-steps of the dynamics, starting from 8 different initial states. *a.* Samples generated in the first experiment, where the TAP approximation was employed in the clamped limit with no inhibition. *b.* Samples generated in the second experiments, where the correlation where registered during the time evolution of the network, and where finite time dependent fields, constrained synapses and the "winner-takes-all" inhibition scheme where considered. The superior smoothness of the samples from the first experiment is also due to the choice of a higher temperature in the dynamics ($\beta_1 = 2$ against $\beta_2 = 30$, see Methods).

Generative tasks

A better way of assessing the quality of the internal representation of the learned dataset in the two experiments is to test the generative properties of the networks. As shown in fig. S2, we obtained some visible configurations from the steady-state distribution of the models, generated according to the information learned from the training images. The steady-state distribution is reached by the dynamical evolution of the network when starting from a visible neuronal state induced by one of the learned images. In order to initialize the network, visible neurons are clamped with a very strong field ($\lambda^{\text{ext}} = 50$) in the direction the image and of the correct label for an initial period of 30 time steps. The field on the first 784 neurons is then removed, while the visible neurons receiving the supervised stimulus are maintained clamped, and the network is left evolving for some iterations. Keeping the output labels clamped only mildly encourages the network to produce new samples from the same category, and this small signal does not have a major effect.

Alternatively the networks can be asked to generate the correct label of a test image, presented to the network with a clamping signal. In the first experiment, the output of the network was read directly from the magnetizations obtained at convergence of the TAP equations iterative procedure, by simply picking the maximum magnetization between the ones corresponding to the visible neurons associated to the label of each category. This network was able to reach a generalization error rate of 2, 76%. This result is far from state-of-the-art classification performance (around 0.3% [21]), but is remarkably low if one takes into account the highly noisy environment and the small size of the network. In the second experiment, the magnetizations were instead explicitly registered during the dynamical evolution of the network. In this case, the performance declined to a 7.74% generalization error rate. This result is nevertheless of interest, considering that the entire learning process was done without a clear supervised signal and that the system was subject to a number of biological requirements restraining the computational performance of the network.

---

[1] Basilis Gidas. Consistency of maximum likelihood and pseudo-likelihood estimators for gibbs distributions. In *Stochastic differential systems, stochastic control theory and applications*, pages 129–145. Springer, 1988.

[2] E Gardner. The space of interactions in neural network models. *Journal of Physics A: Mathematical and General*, 21(1):257, 1988. URL: http://stacks.iop.org/0305-4470/21/i=1/a=030.

[3] Carlo Baldassi, Federica Gerace, Luca Saglietti, and Riccardo Zecchina. From inverse problems to learning: a statistical

mechanics approach. In *Journal of Physics: Conference Series*, volume 955, page 012001. IOP Publishing, 2018.

[4] Andreas Engel. *Statistical mechanics of learning*. Cambridge University Press, 2001.

[5] Alireza Alemi, Carlo Baldassi, Nicolas Brunel, and Riccardo Zecchina. A three-threshold learning rule approaches the maximal capacity of recurrent neural networks. *PLOS Computational Biology*, 11(8):1–23, 08 2015. URL: http://dx.doi.org/10.1371%2Fjournal.pcbi.1004439, doi:10.1371/journal.pcbi.1004439.

[6] Jonathan Binas, Ueli Rutishauser, Giacomo Indiveri, and Michael Pfeiffer. Learning and stabilization of winner-take-all dynamics through interacting excitatory and inhibitory plasticity. *Frontiers in computational neuroscience*, 8:68, 2014. URL: http://journal.frontiersin.org/article/10.3389/fncom.2014.00068/full.

[7] Rodney J Douglas, Kevan AC Martin, and David Whitteridge. A canonical microcircuit for neocortex. *Neural computation*, 1(4):480–488, 1989. URL: http://www.mitpressjournals.org/doi/abs/10.1162/neco.1989.1.4.480#.WCyY6bUc10w, doi:10.1162/neco.1989.1.4.480.

[8] Vernon B Mountcastle. The columnar organization of the neocortex. *Brain*, 120(4):701–722, 1997. URL: http://dx.doi.org/10.1093/brain/120.4.701.

[9] Tom Binzegger, Rodney J Douglas, and Kevan AC Martin. A quantitative map of the circuit of cat primary visual cortex. *The Journal of Neuroscience*, 24(39):8441–8453, 2004. URL: http://dx.doi.org/10.1523/JNEUROSCI.1400-04.2004.

[10] Rodney J Douglas and Kevan AC Martin. Recurrent neuronal circuits in the neocortex. *Current Biology*, 17(13):R496–R500, 2007. URL: http://dx.doi.org/10.1016/j.cub.2007.04.024.

[11] Matteo Carandini and David J Heeger. Normalization as a canonical neural computation. *Nature Reviews Neuroscience*, 13(1):51–62, 2012. URL: http://www.nature.com/nrn/journal/v13/n1/abs/nrn3136.html.

[12] Sebastian Handrich, Andreas Herzog, Andreas Wolf, and Christoph S Herrmann. A biologically plausible winner-takes-all architecture. In *International Conference on Intelligent Computing*, pages 315–326. Springer, 2009. URL: http://link.springer.com/chapter/10.1007/978-3-642-04020-7_34, doi:10.1007/978-3-642-04020-7_34.

[13] Zhi-Hong Mao and Steve G Massaquoi. Dynamics of winner-take-all competition in recurrent neural networks with lateral inhibition. *IEEE transactions on neural networks*, 18(1):55–69, 2007. URL: http://ieeexplore.ieee.org/document/4049830/?arnumber=4049830.

[14] Nancy Lynch, Cameron Musco, and Merav Parter. Computational tradeoffs in biological neural networks: Self-stabilizing winner-take-all networks. *arXiv preprint arXiv:1610.02084*, 2016. URL: https://arxiv.org/abs/1610.02084.

[15] Matthias Oster and Shih-Chii Liu. Spiking inputs to a winner-take-all network. *Advances in Neural Information Processing Systems*, 18:1051, 2006.

[16] Yuguang Fang, Michael A Cohen, and Thomas G Kincaid. Dynamics of a winner-take-all neural network. *Neural Networks*, 9(7):1141–1154, 1996. URL: http://dx.doi.org/10.1016/0893-6080(96)00019-6.

[17] Bertrand Fontaine, José Luis Peña, and Romain Brette. Spike-threshold adaptation predicted by membrane potential dynamics in vivo. *PLoS Comput Biol*, 10(4):e1003560, 2014. URL: http://journals.plos.org/ploscompbiol/article?id=10.1371/journal.pcbi.1003560.

[18] Chao Huang, Andrey Resnik, Tansu Celikel, and Bernhard Englitz. Adaptive spike threshold enables robust and temporally precise neuronal encoding. *PLoS Comput Biol*, 12(6):e1004984, 2016. URL: http://journals.plos.org/ploscompbiol/article?id=10.1371/journal.pcbi.1004984.

[19] Daniel J Amit, Hanoch Gutfreund, and Haim Sompolinsky. Statistical mechanics of neural networks near saturation. *Annals of physics*, 173(1):30–67, 1987. URL: http://www.sciencedirect.com/science/article/pii/0003491687900923, doi:doi:10.1016/0003-4916(87)90092-3.

[20] H. J. Kappen and J. J. Spanjers. Mean field theory for asymmetric neural networks. *Phys. Rev. E*, 61:5658–5663, May 2000. URL: http://link.aps.org/doi/10.1103/PhysRevE.61.5658, doi:10.1103/PhysRevE.61.5658.

[21] Yann LeCun. The MNIST database of handwritten digits. http://yann.lecun.com/exdb/mnist/.

[22] Hugo Larochelle, Michael Mandel, Razvan Pascanu, and Yoshua Bengio. Learning algorithms for the classification restricted boltzmann machine. *Journal of Machine Learning Research*, 13(Mar):643–669, 2012.
